# Rapid flooding-induced adventitious root development from preformed primordia in *Solanum dulcamara*

**DOI:** 10.1093/aobpla/plt058

**Published:** 2013-12-30

**Authors:** Thikra Dawood, Ivo Rieu, Mieke Wolters-Arts, Emiel B. Derksen, Celestina Mariani, Eric J. W. Visser

**Affiliations:** 1 Department of Molecular Plant Physiology, Institute for Water and Wetland Research, Radboud University Nijmegen, Heijendaalseweg 135, 6525 AJ Nijmegen, The Netherlands; 2 Department of Experimental Plant Ecology, Institute for Water and Wetland Research, Radboud University Nijmegen, Heijendaalseweg 135, 6525 AJ Nijmegen, The Netherlands

**Keywords:** Adventitious roots, cDNA-AFLP, gene expression, partial submergence, root primordia, soil flooding, *Solanum dulcamara*, waterlogging.

## Abstract

Flooding is a common stress factor in both natural and agricultural systems, and affects plant growth by the slow diffusion rate of gases in water. This results in low oxygen concentrations in submerged tissues, and hence in a decreased respiration rate. Understanding the responses of plants to flooding is essential for the management of wetland ecosystems, and may benefit research to improve the flood tolerance of crop species. This study describes the response to partial submergence of bittersweet (*Solanum dulcamara*). Bittersweet is a Eurasian species that grows both in dry habitats such as coastal dunes, and in wetlands, and therefore is a suitable model plant for studying responses to a variety of environmental stresses. A further advantage is that the species is closely related to flood-intolerant crops such as tomato and eggplant. The species constitutively develops dormant primordia on the stem, which we show to have a predetermined root identity. We investigated adventitious root growth from these primordia during flooding. The synchronized growth of roots from the primordia was detected after 2–3 days of flooding and was due to a combination of cell division and cell elongation. Gene expression analysis demonstrated that the molecular response to flooding began within 2 h and included activation of hypoxia and ethylene signalling genes. Unexpectedly, these early changes in gene expression were very similar in primordia and adjacent stem tissue, suggesting that there is a dominant general response in tissues during early flooding.

## Abstract (precis)

Flooding strongly affects plant growth, as it leads to low oxygen concentrations in the submerged tissues. Understanding plant responses to flooding may benefit both management of wetland ecosystems and improve progress in creating flood-tolerant crop species. Bittersweet (*Solanum dulcamara*), a species related to tomato and eggplant, has dormant primordia on the stem that develop into adventitious roots within 3 days of flooding. Changes in gene expression were present within 2 hours and included activation of hypoxia and ethylene signalling genes. Unexpectedly, these early changes in gene expression were closely similar in primordia and adjacent stem tissue, suggesting a dominant general response in tissues during early flooding.

## Introduction

Flooding is a significant abiotic stress factor that affects plant growth and development in natural ecosystems (Blom and Voesenek 1996; Visser *et al.* 1996*a*). It severely reduces the exchange of gaseous compounds between the plant and its environment, because gas diffusion is around 10 000-fold slower in water than in air (Armstrong *et al.* 1991). In particular, oxygen deficiency causes poor respiration in submerged plant tissues. This is especially so in organs such as roots when buried in anoxic sediment, resulting in hampered plant growth and survival (Bailey-Serres and Voesenek 2008). Most plants cope with these changes in the short term by changing from aerobic respiration to glycolysis and fermentation for energy production (Bailey-Serres *et al.* 2012). Well-adapted plants, such as many wetland species, also display morphological and growth adaptations that enable them to deal with flooding stress over the longer term. Such adaptions include re-orientation of petioles in a more upright position and faster stem or leaf elongation that enables the shoot to regain contact with the water surface and the open atmosphere (Jackson 1985; Voesenek *et al.* 2004; Fukao and Bailey-Serres 2008). Growth adaptations to submergence have been studied extensively in rice and *Rumex*, showing a pivotal role for the hormone ethylene (reviewed in Voesenek and Sasidharan 2013).

Another common adaptation to flooding is the formation of adventitious roots that contain aerenchyma, i.e. air channels that connect to the shoot and herewith help to maintain gas diffusion under submergence (Evans 2003). Adventitious roots functionally replace primary root systems that may deteriorate during flooding due to oxygen deficiency (Jackson and Drew 1984; Barlow 1986; Sauter 2013), although direct experimental demonstration of their efficacy is still lacking. In some species, the adventitious root primordia are formed *de novo* upon flooding and subsequently continue their development into roots, e.g. in sunflower and in young tomato plants (Wample and Reid 1978; Vidoz *et al.* 2010). In other species, the primordia are constitutively preformed on the stem and stay dormant until stimulated to grow out by flooding. This phenomenon is characteristic of the grass family (Poaceae). In rice, preformed primordia are the source of nodal crown roots that grow out during flooding (Lorbiecke and Sauter 1999). Here, the outgrowth of the adventitious roots is associated with vacuolation and elongation of the root cells in the basal region of the stele (Itoh *et al.* 2005). In rice, the apical meristem in adventitious root primordia is activated by flooding before the emergence of the roots and involves accumulation of cell cycle gene mRNA (Lorbiecke and Sauter 1999). Just before roots emerge, the epidermal cell layers at the apex of the primordia undergo programmed cell death, which appears to be a necessary process to facilitate protrusion of the roots to the outside (Mergemann and Sauter 2000; Steffens and Sauter 2005, 2009; Steffens *et al.* 2012). To test whether or not this flood-responsive developmental pattern is limited only to rice, and whether it occurs more generally in angiosperms, we analysed adventitious root emergence in the dicot species *Solanum dulcamara* (bittersweet).

*Solanum dulcamara* is a diploid Eurasian species of the section Dulcamaroid in the subgenus *Potatoe* of the Solanaceae, which includes the crop species tomato and potato (Weese and Bohs 2007; D'Agostino *et al.* 2013; Knapp 2013). The species occupies a wide range of ecologically contrasting habitats (Horvath *et al.* 1977) but prefers sites that are wet throughout the year and often flooded. It is therefore commonly found along river banks, canals, ditches, and in mires and damp woods (Pegtel 1985). However, it can also grow in sandy soil on the slopes of primary coastal dunes (T. Dawood, C. Mariani and E. J. W. Visser, pers. observ.). The main stem and branches of *S. dulcamara* typically carry numerous adventitious root primordia, derived from the parenchyma of the rays in the xylem, where cells situated near the phloem region undergo cell division to make up a conical base that connects with the vascular bundle (Terras 1897). Importantly, the fully developed primordia are clearly visible and easily accessible, making *S. dulcamara* an excellent model system to analyse the developmental and signalling processes taking place during primordium activation and early root growth.

In this study, the floodwater was deep enough both to flood the original roots and submerge the lower part of the stem of the *S. dulcamara* plants. From the latter region, we sampled preformed adventitious root primordia at different time intervals after the onset of flooding to determine the timing of events preceding outgrowth of the roots. This was done by histological analysis and complementary DNA-amplified fragment length polymorphism (cDNA-AFLP) profiling to reveal early responsive genes. The minimum period during which the lower part of a plant needed to be submerged to develop adventitious roots was also determined.

## Methods

### Plant material and growth conditions

Seeds of *S. dulcamara* wild-type plants growing in a wet habitat near Wychense Ven (Wijchen, The Netherlands) were collected in 2005 and stored at 4 °C by the Experimental Garden and Genebank (Radboud University, Nijmegen, The Netherlands) (accession no. A54750008). Seeds from this seed stock were sown in vermiculite in small round plastic pots 10 cm tall and 13 cm in diameter, kept in the dark at 4 °C for 3 days and then grown in a greenhouse, with a daily temperature regime of 20–23 °C (day) and 15–18 °C (night), with additional light supplied by high-pressure sodium lamps (SON-T; 600 W; Philips Nederland B.V., Eindhoven, The Netherlands). Three-week-old seedlings were individually transplanted into 12 × 11 × 11 cm (h × w × d) plastic pots filled with potting soil (Stekgrond, Holland Potgrond, Grubbenvorst, The Netherlands) and kept further under the same conditions. The plants were watered daily and fertilized once every 2 weeks until 10–12 weeks old, when they were experimentally treated. By 8 weeks, the position of primordia gradually became visible as small bumps on the epidermis of the stem.

To isolate the primordia samples, external excisions were made around the primordia, which were distinguishable by their white dome-shaped structure. This was followed by peeling off of the primordia while minimizing contamination with surrounding stem tissue. To isolate stem samples, similar sections were taken from green stem tissue in which no primordia were present.

### Flooding and high-humidity treatments

About 15 primordia on the basal section of the stem were marked with a marker pen 2 or 3 days prior to the treatment of the plants. One day before treatment, glass containers (60 × 21 × 21 cm, h × w × d) were filled with tap water to a level that would allow flooding of the plants to 15 cm above the soil surface. Plants were placed individually in the containers at 1100 h. After the plants were flooded, emergence of the adventitious roots was scored by eye every day for 1 week. Untreated control plants were placed in identical containers without floodwater. A localized flooding experiment was conducted by enclosing a randomly chosen stem section containing primordia in a glass cuvette containing water and sealing water-tight with Terostat Butyl-IX (Henkel AG & Co., Düsseldorf, Germany).

To create a high-humidity treatment, glass containers similar to those used for flooding were lined with wet filter paper (standard filter paper; Schleicher and Schuell GmbH, Dassel, Germany). Then plants were placed individually in the containers and the open top was covered with plastic film. During this treatment, air was flushed at 1.75 L min^−1^ through each glass container to prevent accumulation of plant-derived volatiles such as CO_2_ or ethylene. The flushing air was first humidified by directing it through a water column. Humidity in the containers was measured with a thermohygrometer (type 605-H1, Testo AG, Lenzkirch, Germany) and showed levels of 90–93 % relative humidity. Control plants were kept in similar but open containers and flushed with dry air. Light and temperature were kept at standard conditions (see above). In one experiment, flooding treatment for 2 days was followed by transfer to humid air. Adventitious roots were counted 7 days after the start of the experiment.

### Histological analysis and microscopy

Dissected primordia were fixed for 2 h in 2 % glutaraldehyde in 0.1 M phosphate buffer (pH 7.2), rinsed in buffer for 2 h and then treated with 1 % (w : v) osmium tetroxide solution overnight at 4 °C. Subsequently, the primordia were dehydrated in 100 % ethanol and embedded in Spur's medium. Sections of 1 µm were stained with aqueous toluidine blue solution (0.1 % (v : v) in 1 % (w : v) borax) and viewed under a Leitz Orthoplan microscope with a Leica DFC420C camera (both Leica Microsystems B.V., Rijswijk, The Netherlands). For each time point of the experiment (0, 24, 48 and 72 h after the onset of flooding), median and transverse sections of four primordia were examined. The area of the meristematic region (mm^2^) included cells containing at least two-thirds cytoplasm, as identified by dense staining with toluidine blue. The size of meristematic cells (µm^2^) was determined in a projected oval around the quiescent centre (QC). To determine the number of meristematic cortical cells in the proximal–distal direction, numbers were counted in the cortical rows right and left of the vascular bundle in the primordia.

### Starch staining

Primordia were dissected after 0, 24 and 48 h of flooding, sectioned in 100 µm pieces with a vibrating microtome (Leica VT1000 S; Leica Biosystems, Rijswijk, The Netherlands) and placed directly in Lugol's solution (1 g of iodine and 2 g of potassium iodide dissolved in 300 mL water) for 3 min. Thereafter, sections were kept for 3 days in the clearing agent chlorallactophenol as described in Herr (1993). Imaging was done on a Leitz Orthoplan (see above).

### DR5:GUS construct and plant transformation

The DR5:GUS construct (Ulmasov *et al.* 1997) was transformed to *Agrobacterium tumefaciens* strain GV3101 using freeze–thaw transformation (Chen *et al.* 1994). Transgenic plants were generated by the leaf disc transformation method. In short, leaves were harvested from 3- or 4-week-old *S. dulcamara* plants, sterilized for 10 min in a solution of 1.5 % bleach and 0.01 % Tween 20 (v : v), and washed four times for 5 min with sterilized demineralized water. The leaf explants were cut without veins and incubated in a 1 : 100 diluted bacterial culture (OD_600_ 0.4–0.6; diluted with liquid MS20 co-cultivation medium consisting of 20 g L^−1^ sucrose, 4.4 g L^−1^ Murashige and Skoog (MS) with Gamborg B5, 0.5 g L^−1^ 2-(*N*-morpholino)ethanesulfonic acid monohydrate pH 5.8, 2 mg L^−1^ 6-benzylaminopurine (BAP), 0.1 mg L^−1^ 1-naphthaleneacetic acid (NAA) and 10 mg L^−1^ acetosyringone) and kept packed in aluminium foil for 3 days under standard climate chamber growth conditions (16/8 h day/night, at 20/22 °C). Thereafter, leaf explants were transferred to a selective medium of MS20 supplemented with growth regulators (2 mg L^−1^ BAP, 0.1 mg L^−1^ NAA, 300 mg L^−1^ cefotaxime, 300 mg L^−1^ vancomycin and 25 mg L^−1^ kanamycin). The plates were covered with three layers of filter paper and kept for a week in a standard climate chamber. The filter papers were removed gradually (one per week). Every two and a half weeks, the explants were transferred to fresh selective medium. After ∼7 weeks, newly emerged shoots were excised and transferred to MS20 medium supplemented with 300 mg L^−1^ cefotaxime, 300 mg L^−1^ vancomycin, 10 mg L^−1^ kanamycin and 0.25 mg L^−1^ indole-3-butyric acid (IBA). When roots had formed, plants were transferred to the greenhouse.

### β-Glucuronidase staining

Histochemical β-glucuronidase (GUS) staining was performed on 50-µm-thick dissected primordia from DR5:GUS transgenic plants after 0, 24 and 48 h of flooding. β-Glucuronidase activity was tested by incubating sections of the primordia overnight at 37 °C under dark conditions in GUS staining buffer, containing 10 mM EDTA, 0.1 % (v : v) Triton X-100, 0.5 mM ferricyanide, 0.5 mM ferrocyanide and 2 µg L^−1^ X-Gluc, in 50 mM Na-phosphate buffer, pH 7.5. Sections were cleared and stored in 70 % (v : v) ethanol.

### cDNA-AFLP

Primordia and stem explants were dissected from the flooded plants at 0, 6, 12, 24, 48 and 72 h after flooding, and frozen directly in liquid nitrogen. Three biological replicates were collected for each time point. Total RNA was isolated from the frozen primordia samples with Trizol (Invitrogen, Carlsbad, CA, USA). PolyA^+^-RNA was captured from 5 µg of total RNA using biotinylated oligodT-coated streptavidin beads (Dynabeads^®^ MyOne™ Streptavidin C1; Invitrogen), according to the manufacturer's protocol. First and second strands of cDNA were synthesized as described by Vriezen *et al.* (2008). Double-stranded cDNA was used for cDNA-AFLP and the liberated BsYI–MseI fragments were used for the next steps of this procedure as described by Bachem *et al.* (2008). Gene expression in the RNA samples was covered using 128 primer combinations for selective amplification as described by Breyne *et al.* (2002). This was estimated to cover circa 60 % of the transcriptome. For a list of primers **[see Supporting Information]**. We selected 114 fragments ranging from 34 to 582 bp.

### Characterization of cDNA-AFLP fragments

To identify the genes represented by the transcript-derived fragments (TDFs), two independent fragments of each differentially expressed gene were cut out from the gel and DNA was re-amplified under the same conditions as for selective amplification. Subsequently, the fragments were blunted and cloned in pJET 1.2/blunt cloning vector (Fermentas, Thermo Scientific Molecular Biology Solutions, Landsmeer, The Netherlands) and sequenced using the Beckman DTCS quick start mix, cat.# 608120 (Beckman Coultier, Brea, CA, USA), and the Beckman CEQ™ 2000 DNA analysis system. The sequences that corresponded to the correct size of the cDNA-AFLP fragments were aligned to the *S. dulcamara* transcriptome assembly (D'Agostino *et al.* 2013), the tomato transcript database (ITAG2.3; http://www.sgn.cornell.edu) and the *Arabidopsis* transcript database (TAIR10), using BLASTn with an *E* value <1 × 10^−10^.

From the total 114 TDFs, 22 were discarded because multiple different sequences were obtained from the fragment and 11 were excluded because they had the same hit in tomato as another TDF, but at a different position. This might indicate a multiple enzymatic restriction in the same transcript. Among the 81 TDFs that were considered to be the representative sequences for our analysis, 37 were up-regulated and 44 down-regulated **[see** **Supporting Information****]**. We found significant homology for 72 of these TDFs; only nine fragments, ranging from 34 to 222 bp, did not show any homology. These were further blasted to the tomato genome (version SL2.40), but only one of them, namely TDF 22-5, could be aligned to the intron region of *Solyc12g008710*. Our not finding any homology for the other eight TDFs might be explained by the small size of the fragments or because they represent less conserved regions of transcripts. For several TDFs, we could not obtain a hit in the *S. dulcamara* transcriptome assembly, even though a hit was found in the tomato database **[see** **Supporting Information****]**.

### Real-time quantitative reverse transcription polymerase chain reaction

Primordia and stem tissues were dissected from flooded and control plants at 2, 4, 6, 12 and 24 h after flooding in two biological replicas. Control samples were taken at the same time of the day to avoid the influence of a circadian rhythm. For this experiment, 10- to 12-week-old plants were used and prepared as described above. Each sample was a pool of two plants to minimize biological variation. Total RNA was isolated from the frozen primordia and stem tissues using the RNeasy Plant Mini Kit (QIAGEN, Hilden, Germany). Contaminating genomic DNA was removed using DNase I, RNase-free (Fermentas, Thermo Scientific Molecular Biology Solutions). The total DNA-free RNA (250 ng) was then reverse-transcribed using a cDNA synthesis kit (iScript™ cDNA synthesis kit; Bio-Rad Laboratories B.V., Veenendaal, The Netherlands) in a reaction volume of 20 µL. For quantitative polymerase chain reaction (qPCR), 5 µL of 10-fold diluted cDNA were used in a 25-µL PCR reaction containing 400 nM each primer and 12.5 µL of iQ™ SYBR Green Supermix (Bio-Rad Laboratories B.V.). The PCR reactions were performed in a 96-well thermocycler (Bio-Rad iCycler) by starting with 3 min at 95 °C followed by 45 cycles consisting of 15 s at 95 °C and 60 s at 60 °C. Melt curves were generated by raising the temperature from 65 to 95 °C in 0.5 °C increments per 10 s to verify the presence of a specific product. Expression of the *S. dulcamara* homologues of the tomato genes *TIP4 I*, *SAND*, *CAC* and *Expressed* (Expósito-Rodrìguez *et al.* 2008) was used as the endogenous control. The stability of the reference genes was evaluated with geNorm software. The transcripts level was quantified as described in Rieu and Powers (2009). The primers used for real-time quantitative-PCR were *TIP4 I*: Forw: 5′-AGTCATGCCTAGTGGTTGGTTCC-3′; Rev: 5′-TGAGCACTCCATCAACCCTAAGC-3′; *SAND*: Forw: 5′-TGCTTACACATGTCTTCCACTTGC-3′; Rev: 5′-AAACAGGACCCCTGAGTC AGTTAC-3′; *Exp*: Forw: 5′-CTAAGAACGCTGGACCTAATGACAAG-3′; Rev: 5′-AAAGTCGATTTAGCTTTCTCTGCATATTTC-3′; *CAC*: Forw: 5′-AGTTTGTTGTTGAGGCTGTTACAC-3′; Rev: 5′-ACCGGACACCTTCCTGAGTAATG-3′; *21-8*: Forw: 5′-AGACGAGCATCGCCTGAACC-3′; Rev: 5′-GCTGCAACAATGGCTTTTCTTCTC-3′; *16-1*: Forw: 5′-GCACATTCACTACA AAACTTTCAGACTC-3′; Rev: 5′-TAGTTGTGAAATCTTCGAGGAGATGTTG-3′; *21-10*: Forw: 5′-CGGGTCTGAGTCAACAGAGGAC-3′; Rev: 5′-TCATCCAGCTTCAACGGTGAGG-3′; *18-14*: Forw: 5′-GATCCCAATAAAGGGTGGCGAATC-3′; Rev: 5′-TGCTGGTGCCTCCTCCAAAC-3′; *17-6*: Forw: 5′-TGAGGAAGAGAGGGAGCAATAAGTG-3′; Rev: 5′-GCAGCAAACTTTATTC CATAATACATAGTG-3′; *17-4*: Forw: 5′-GGATTGTTATGTGTTCAAGAAGATGTCAG-3′; Rev: 5′-AGTGGTGATAGAGTAACTATTAAGCATGAG-3′.

### Statistical analysis

All data were log transformed before analysis to correct for heterogeneity of variance (Gomez and Gomez 1984; Rieu and Powers 2009). One-way analysis of variance with uncorrected least significant difference was applied to assess the significance of differences between flooding and the control condition, using SPSS, version 18 (IBM, New York, NY, USA).

## Results

### Adventitious root emergence in *S. dulcamara* upon flooding

**Figure 1.**
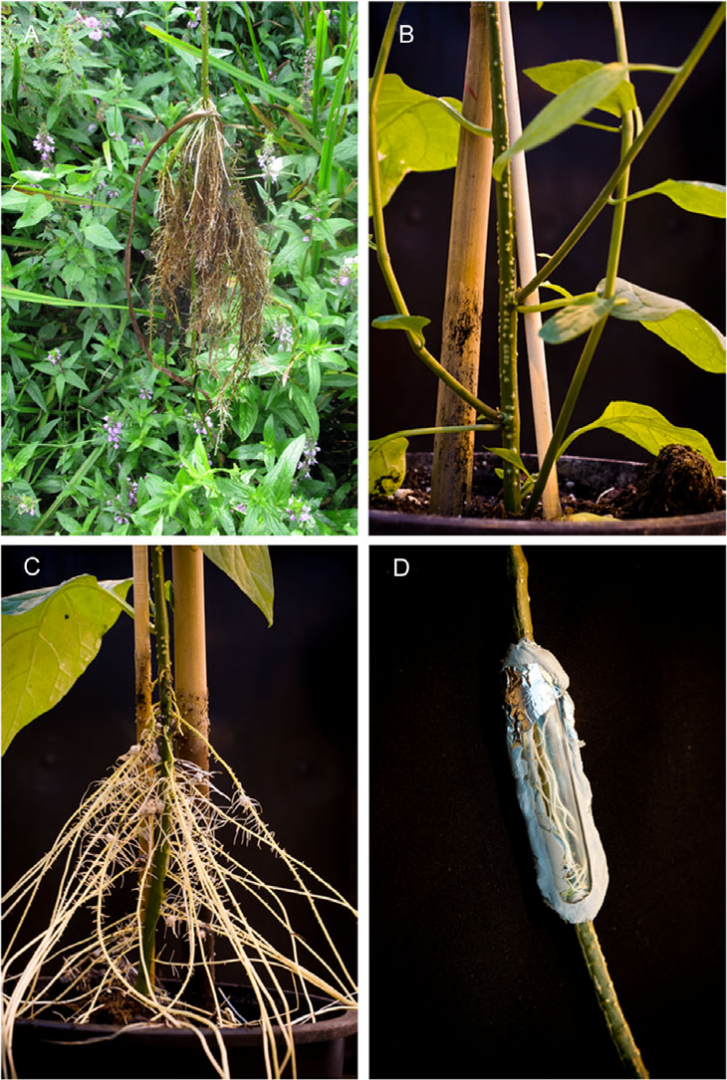
Adventitious root formation on stems of *S. dulcamara* during flooding. (A) Adventitious roots grown on a plant during a flood in a natural floodplain. (B) Adventitious root primordia on the stem of a plant grown under greenhouse conditions prior to flooding. (C) Adventitious roots grown after flooding in the greenhouse. The water level was kept at about 15 cm above the soil (blue bar) for 2 weeks and then removed for photography. (D) Adventitious root growth on a locally flooded stem section. The stem of the intact *S. dulcamara* plant was surrounded with water in a sealed cuvette for 1 week. Adventitious roots developed only on the stem part that was inside the cuvette.

**Figure 2.**
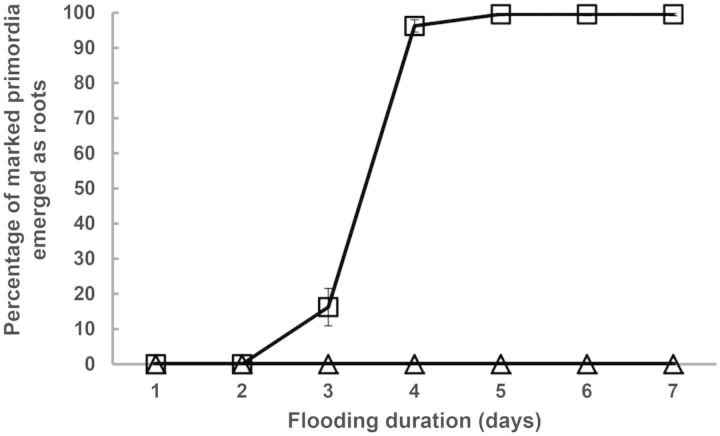
Rate of adventitious root emergence in *S. dulcamara* during flooding. Plants were partially submerged or kept in air and root emergence was scored every day by eye for 1 week after the start of the treatment. The mean percentage of primordia grown out into adventitious roots (±SE) is indicated (*n* = 12 plants for each treatment).

In natural floodplains, we observed that *S. dulcamara* developed many shoot-borne adventitious roots upon flooding of the roots and submergence of the stem base during a natural flood (Fig. 1A). Adventitious root primordia were constitutively present on the stem and internodes of this species (i.e. preformed; Fig. 1B; Terras 1897) and developed into adventitious roots when the stem was submerged for several days in the greenhouse (Fig. 1C). Even when flooding was applied to only a section of the stem (achieved by sealing a part of the intact stem in a glass cuvette filled with water), adventitious roots emerged, indicating that their induction is independent of flooding of the primary root system (Fig. 1D). In both cases, the response was strictly local, as primordia that remained dry never developed into a root (Fig. 1C and D). Adventitious root growth was shown to be very fast and synchronized, since emergence of all adventitious roots, as scored by eye, already occurred between the second and fourth day after the onset of the flooding treatment (Fig. 2).

### Changes at the histological level during early adventitious root growth

**Figure 3.**
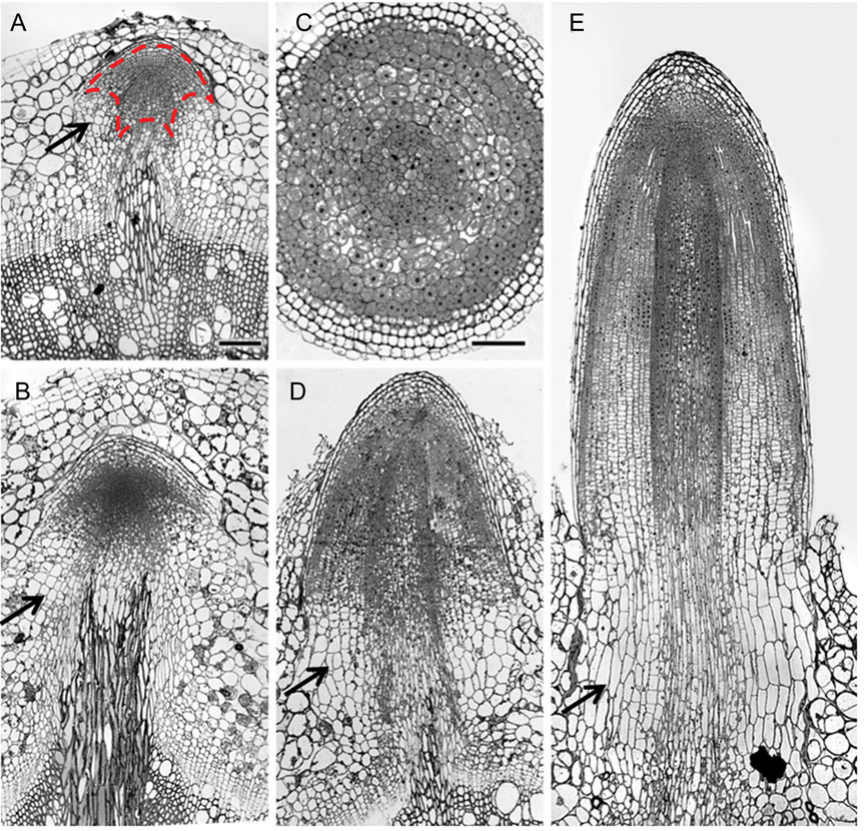
Histological analysis of adventitious root primordia of *S. dulcamara* during flooding. (A) Non-flooded primordium; the dashed red line represents the area of the meristematic region (mm^2^), which includes the densely stained and non-vacuolated cells of the root tip; (B) primordium flooded for 24 h; (C) cross-section and (D) longitudinal section of primordia flooded for 48 h; (E) emergence of the adventitious root after 72 h of flooding. Arrows indicate the parenchymatic cortex cells at the base of the primordium that elongate. The scale bar in (A) also applies to (B), (D) and (E) and represents 100 µm. The scale bar in (C) is 50 µm.

**Figure 4.**
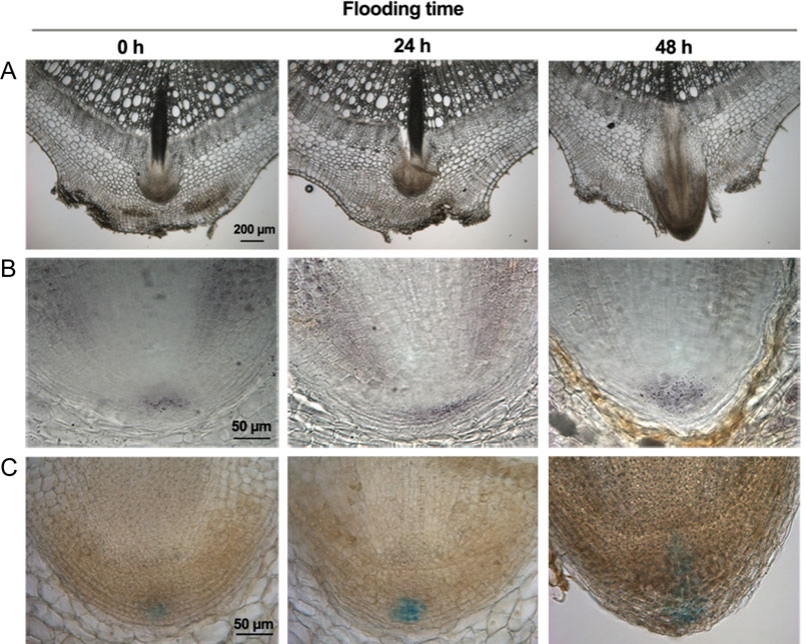
Root meristem identity markers in the adventitious root primordia. (A) Representative longitudinal sections of primordia flooded for 0, 24 and 48 h are shown; (B) Lugol's staining marks starch granules in the differentiated columella cells; (C) expression of the *DR5-GUS* auxin reporter gene in the distal domain of the meristem.

Primordia from flooded and non-flooded plants were dissected and sectioned to analyse changes in the transition from primordium to emerging root (Fig. 3). Preformed non-flooded primordia were dome-shaped structures, with a layered organization, reminiscent of a lateral root (Fig. 3A). We analysed two root meristem markers, columella starch granules (Blancaflor *et al.* 1998) and the maximum of auxin as visualized by GUS staining in *DR5*::*GUS* transgenic plants (Sabatini *et al.* 1999). Both markers confirmed that the primordia already had root identity in their dormant state (Fig. 4B and C). After 24 h of flooding, the structure and the size of the primordia were still similar to those of dormant primordia and the stem tissue around them remained intact (Fig. 3A and B). At 48 h, in contrast, differentiation of different root cell types could be seen. For example, the vascular cylinder was clearly delineated and aerenchyma formation had started (Fig. 3C). At this time, the cortex of the surrounding stem had ruptured, giving way to a protruding root tip (Fig. 3D). Three days after flooding, a fully differentiated short root had emerged from the stem (Fig. 3E). To understand the role of cell division and cell elongation in the emergence process, a zone of meristematic cells was defined as primordium cells rich in cytoplasm (Fig. 3A). Two days after the onset of flooding, the area of the meristematic region had grown significantly larger (Fig. 3D, Table 1). This was due to cell division combined with limited cell expansion, because the size of the cells in the meristematic region was stable, while their number (expressed as the number of cells in the cortex in the proximal–distal direction) had increased (Table 1). Although we did not detect an early increase in cell size in the meristematic part of the primordium, root emergence was associated with elongation of the cortex cells at the base of the primordia. This was clearly detectable after 48 h from the start of flooding (see the arrows in Fig. 3).

**Table 1.** Cytological changes in adventitious root primordia of *S. dulcamara* during flooding. Analysis was performed on longitudinal sections of the primordia. Values indicate the mean (SE in parentheses). *Significantly different from 0-h flooding, *P* < 0.01.

Duration of flooding (h)	*n*	Area of the meristematic region (mm^2^)	Size of meristematic cells (µm^2^)	No. of meristematic cortex cells in the proximal–distal direction
0	4	0.061 (0.013)	102 (3.7)	17 (1.7)
24	4	0.073 (0.005)	116 (8.5)	15 (0.9)
48	3	0.196 (0.027)*	113 (9.8)	29 (3.0)*
72	1	0.441*	102	80*

In conclusion, flooding induces both division and elongation of the primordia cells, leading to emergence of adventitious roots with a mature internal root anatomy becoming evident after 48 h flooding.

### cDNA-AFLP profiling reveals a rapid molecular response to flooding

**Figure 5.**
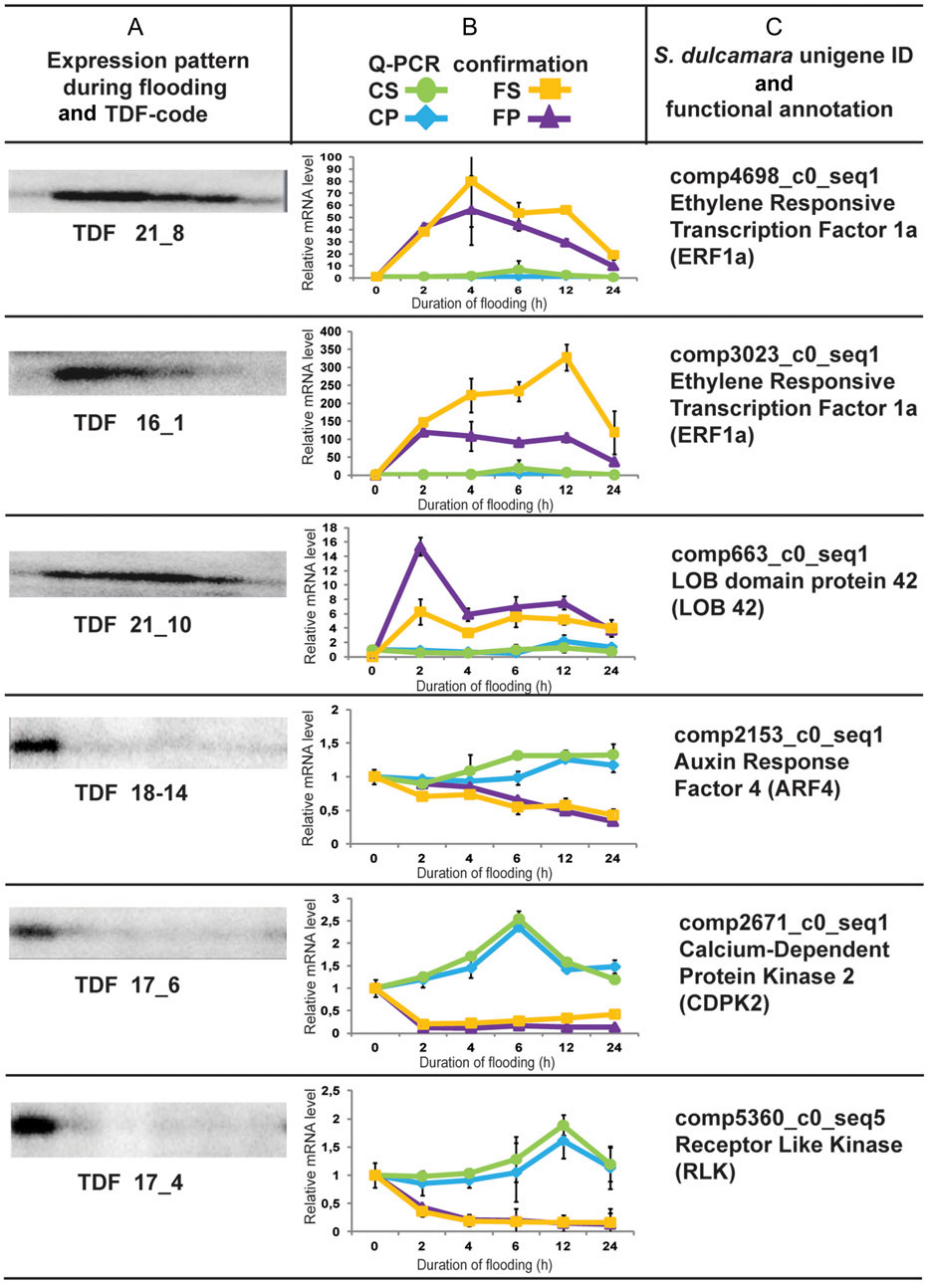
Expression of transcripts selected from cDNA-AFLP analysis of the flooding response. Column (A) shows the expression patterns obtained by cDNA-AFLP for the selected TDFs at 0, 6, 12, 24, 48 and 72 h after flooding. Column (B) shows the relative mRNA level of the transcripts corresponding to the TDFs in flooded stem (FS), flooded primordia (FP), control stem (CS) and control primordia (CP) at the time points 0, 2, 4, 6, 12 and 24 h after flooding. Data are means ± SE, *n* = 2. Column (C) shows the corresponding *S. dulcamara* unigene ID (D'Agostino *et al.* 2013) and annotation.

To detect early changes in gene expression associated with the development of adventitious roots from preformed primordia, we analysed the transcript profile of primordia-enriched tissue samples up to 72 h after the start of treatment using cDNA-AFLP. Differentially expressed TDFs were identified based on clear visual differences in intensity between time points and on consistency of the observed changes in all replicas (Fig. 5A). In total, we identified 114 differentially expressed TDFs representing either up- or down-regulated transcripts, as compared with the level of expression at time zero **[see** **Supporting Information****]**. Most of the transcripts revealed by this analysis were modulated in abundance after only 6 h of flooding, and in the case of the up-regulated TDFs, their expression decreased again after 72 h (data not shown). Following sequencing, the functional description of the TDFs was performed by aligning them to the published *S. dulcamara* transcriptome (D'Agostino *et al.* 2013). The identified TDFs were then classified into biologically functional categories by considering the annotation data and literature **[see** **Supporting Information****]**. Among the up-regulated transcripts, we found genes involved in ‘transcription regulation’ and some of these were related to the ‘ethylene response’. Genes belonging to categories of ‘signalling’, ‘protein synthesis and metabolism’ and ‘carbohydrate metabolism and glycolysis’ were also changed. Among the down-regulated genes, similar gene categories could be recognized, although no genes related to ethylene response and carbohydrate metabolism and glycolysis were present, while a group of genes related to ‘cell wall’ modification could now be identified.

To improve our understanding of the timing of the early signalling events involved in primordia activation, we studied in more detail the expression of a subset of transcripts from the ‘signalling’ and ‘transcription regulation’ categories. Samples were taken at shorter time intervals up to 24 h after flooding, and from both primordia and adjacent stem tissue. Non-flooded control samples were taken in parallel to correct for the influence of a circadian rhythm. In general, the qPCR results showed that the expression of the analysed genes was modified very early during flooding, starting within 2 h (Fig. 5B). For most of the up-regulated genes, the change in expression was transient, with expression of the LOB domain protein 42 transcript (TDF 21-10) peaking within 2 h. Also, the modulation of down-regulated genes started after 2 h with the exception of auxin response factor (ARF4), which decreased more gradually during flooding. Notably, *ERF1a* (TDF 16-1) was very strongly up-regulated, increasing almost 350-fold in the stem after 12 h. Furthermore, the qPCR analysis showed that all six transcripts, either up- or down-regulated, were expressed with a similar pattern in both the stem and primordia (Fig. 5B), and thus no primordia-specific changes in gene expression were detected.

### Primordia activation starts within 24 h of flooding

**Figure 6.**
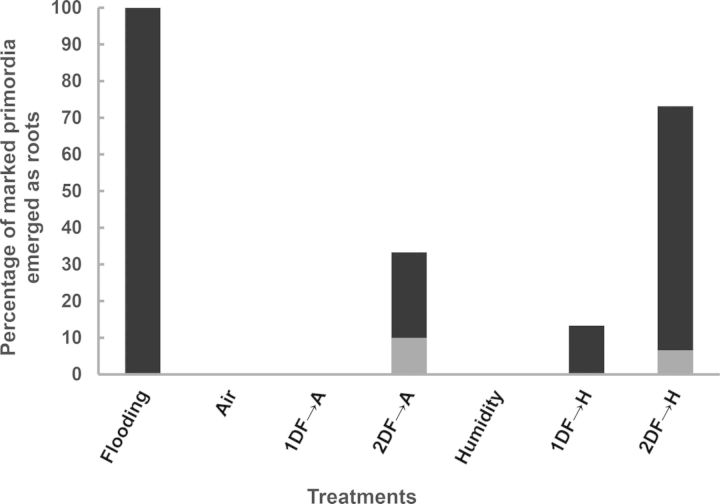
Time needed for activation of preformed primordia in *S. dulcamara* under flooding to emerge into adventitious roots. Plants were treated with continuous flooding or in air for 7 days, exposed to high humidity, or first flooded for 1 (1DF) or 2 days (2DF) and then transferred to humid air (H) for the remainder of the 7 days. Grey indicates the percentage of primordia grown out into adventitious roots at the switch of treatment; black indicates the percentage of primordia grown out into adventitious roots after 7 days.

Although cytological changes were first observed after 48 h of flooding, molecular analysis showed strong transcriptional re-programming in the first 24 h. To test the hypothesis that the flooding-dependent activation signal is perceived as soon as after 24 h of flooding, we flooded plants for 24 and 48 h, and observed the subsequent adventitious root emergence, in humid air (Fig. 6). While humid air alone did not result in adventitious root outgrowth, 15 % of adventitious roots emerged in the days following a 24-h flooding treatment. After a 48-h treatment, some primordia had already emerged under water and many more emerged in the subsequent days under humidity. Together this shows that a subset of the primordia is already activated within 24 h of flooding and that most of them are activated after 48 h.

## Discussion

Formation of preformed primordia in stems of *S. dulcamara* was first described more than a century ago (Terras 1897). The occurrence of these primordia, with their distinctive dome shape on the stem, is important in vegetative propagation, as roots may grow out from these primordia when parts of the slender stem touch wet soil (Terras 1897). We show that the preformed primordia of this species also grow into adventitious roots upon flooding, suggesting a function as an adaptation to flooding of the soil when the water also covers the lower stem. When the normal roots are flooded and lose contact with the atmosphere, they commonly die due to the anoxic conditions that prevail in flooded soil (Ponnamperuma 1984). Indeed, we observed that the original root system of *S. dulcamara* deteriorates quickly upon flooding (data not shown), making the newly formed adventitious roots, with their aerenchymatous connections linking root apices with the shoot, likely to be important in replacing the former roots.

### Early adventitious root growth is driven by cell division and cell elongation

The formation of adventitious and lateral root primordia involves cell de-differentiation and then division to organize the cell layers from which the root meristem develops (Malamy and Benfey 1997; Itoh *et al.* 2005). In lateral roots, the developmental process continues with cell division to establish the primordia and is then directly followed by cell elongation to propel the root emergence (Malamy and Benfey 1997). In *S. dulcamara*, where the established primordia normally remain dormant, their activation involves cell divisions coupled with cell expansion to increase the size of the meristematic region, followed by cell elongation at the basal region to push the apex radially outwards. Together, these processes drive root emergence. This sequence of events is analogous to the situation in deepwater rice, where the expression of cell cycle genes precedes adventitious crown root emergence upon flooding and ethylene treatments (Lorbiecke and Sauter 1999), and cell elongation occurs in the basal region of the primordia at the time of root emergence (Itoh *et al.* 2005). Whether growth of the adventitious root through the surrounding tissue involves enzymatic weakening of the cell walls, such as during lateral root development in *Arabidopsis* (Péret *et al.* 2009), and programmed death of the epidermal cells, such as in rice (Steffens *et al.* 2012), is not clear as yet.

### Adventitious root primordia are rapidly activated by flooding

A rapid outgrowth of adventitious roots from preformed primordia is consistent with the function of these roots in survival under flooding. Anoxic conditions develop within several hours in a flooded soil (Ponnamperuma 1984), and this might not only be fatal for the original non-aerenchymatous roots, but also inhibit the formation of replacement roots within the soil. In contrast, the stem base will have ready access to oxygen, thus permitting adventitious root formation above the soil. If *de novo* initiation of root primordia was required for this, it might be too slow to have adaptive value. However, outgrowth of preformed primordia, as in *S. dulcamara*, would be much faster. In other flood-adapted species, flooding- or submergence-induced adventitious root growth from preformed primordia can be detected within a few hours, e.g. after 10 h in deepwater rice (Lorbiecke and Sauter 1999), to a few days, e.g. in *Rumex palustris* (Visser *et al.* 1996*a*). Our results showed that in *S. dulcamara* the emergence of these roots started to be visible after 2–3 days from the onset of flooding. Molecular analysis and short-term flooding experiments, however, indicated that the response to flooding started much faster and some of the primordia were irreversibly activated within 24 h. The variation in time of emergence of adventitious roots may thus be explained by the variation in the time it takes for the individual primordia to perceive the ‘flooding signal’ and get activated. In rice, it has been hypothesized that the differences in emergence time are caused by the differences in the age of the nodal primordia before the start of submergence (Lorbiecke and Sauter 1999). As we also noticed variation in the size of the root meristems between primordia, it is possible that the older, slightly larger primordia get activated more easily. Furthermore, the idea that each primordium perceives the activation signal autonomously is consistent with our observation that the response is strictly local: non-flooded primordia never get activated.

### Signalling for primordium activation

What signalling pathways could be involved in adventitious root activation? Hypoxia commonly induces changes in the expression of genes related to carbohydrate metabolism (Zhang *et al.* 2006; Lasanthi-Kudahettige *et al.* 2007; Christianson *et al.* 2010). Also in *S. dulcamara* genes belonging to the category ‘glycolysis’ were rapidly up-regulated, after 6 h of flooding, indicating a metabolic adjustment to flooding stress and suggesting that hypoxia signalling is taking place. In the category ‘transcription regulation’, we found three genes encoding ethylene response factors (*ERF1a*, *ERF2b*) to be up-regulated, suggesting involvement of ethylene signalling. Ethylene has been associated with flooding responses in many species, including the outgrowth of adventitious root primordia (Jackson *et al.* 1981; Visser *et al.* 1996*b*), and ERFs have been found to participate in several morphological and metabolic adaptations. For example, the ERF-type genes *SNORKEL1* and -*2* are up-regulated in deepwater rice varieties within 3 h after submergence (Hattori *et al.* 2009) and confer tolerance to flooding with gradually increasing depth by stimulating the growth of internodes and thus stem elongation to above the water surface. On the other hand, a mutated ERF (*SUB1A*) prevents ethylene-promoted elongation in rice, thereby effecting energy conservation (Xu *et al.* 2006) and conferring tolerance to deep transient floods that cannot be overcome by shoot elongation.

Interestingly, the detailed characterization of six differentially expressed TDFs showed that their transcriptional response, whether up- or down-regulated, was highly similar in primordia and stem. This can imply that a large part of the transcriptional reprogramming which is needed to cope with flooding is independent of tissue type. Activation of primordia may therefore depend more on a tissue-specific response to the common signals present in all plant parts during flooding.

We conclude that *S. dulcamara* is a suitable model system to study activation of dormant adventitious root primordia by flooding. Physiological and molecular analysis in combination with genetic modification are planned to shed further light on the roles of the various signalling pathways.

## Sources of Funding

This research was supported by the Netherlands Organization for Scientific Research (NWO) under project number 017.005.041.

## Contributions by the Authors

T.D., I.R., C.M. and E.V. designed the experiments and wrote the paper; T.D., I.R., E.V., M.W.-A. and E.D. performed the experiments and analyses.

## Conflicts of Interest Statement

None declared.

## Supporting Information

The following Supporting Information is available in the online version of this article –

**Table S1.** List of the up- and down-regulated transcript-derived fragments obtained from the cDNA-AFLP data.

Additional Information
