# Supplementary material for: Rapid flooding-induced adventitious root development from preformed primordia in Solanum dulcamara
Source: AoB Plants. 2013 Dec 30;6:plt058. doi: 10.1093/aobpla/plt058 (PMC3922303; doi:10.1093/aobpla/plt058)
Supplement: Additional Information [file supp_6_plt058_index.html]

Rapid flooding-induced adventitious root development from preformed primordia in Solanum dulcamara — Rapid flooding-induced adventitious root development from preformed primordia in Solanum dulcamara — Additional Information 

# Rapid flooding-induced adventitious root development from preformed primordia in *Solanum dulcamara*

## Additional Information

Additional Information

**Files in this Data Supplement:**

- Additional Information - docx file
