# Supplementary material for: Rapid flooding-induced adventitious root development from preformed primordia in Solanum dulcamara
Source: AoB Plants. 2013 Dec 30;6:plt058. doi: 10.1093/aobpla/plt058 (PMC3922303; doi:10.1093/aobpla/plt058)
Supplement: Additional Information [file supp_plt058_plt058supp.docx]

**SUPPORTING INFORMATION**

**Table S1. Up- and down-regulated TDFs obtained from cDNA-AFLP data.**

| **Up-regulated TDFs** | |  |  |  |
| --- | --- | --- | --- | --- |
|  |  |  |  |  |
| **TDF** | **Dulc unigene** | **Length** | **Functional description (manual)** | **Category Functional** |
| 11_1 | comp131_c0_seq1 | 162 | Phosphoglycerate kinase | Carbohydrate metabolism/ glycolysis |
| 20_5 | comp279_c0_seq1 | 136 | Diphosphate--fructose-6-phosphate 1-phosphotransferase |  |
| 24_2 | comp112_c0_seq1 | 265 | Phosphoenolpyruvate carboxykinase |  |
| 11_9 | comp5145_c0_seq1 | 177 | Myb-related transcription factor | Transcription regulation |
| 16_1 | comp3023_c0_seq1 | 75 | Ethylene responsive transcription factor 1a |  |
| 2_2 | comp414_c0_seq1 | 82 | Ethylene responsive transcription factor 2b |  |
| 21_10 | comp663_c0_seq1 | 148 | LOB domain protein 42 |  |
| 21_8 | comp4698_c0_seq1 | 273 | Ethylene responsive transcription factor 1a |  |
| 25_2 | comp16211_c0_seq1 | 344 | Homeodomain-like superfamily protein |  |
| 3_5 | comp6283_c0_seq1 | 129 | BHLH transcription factor |  |
| 18_12 | comp2971_c0_seq1 | 122 | phototropin 2 | Signalling |
| 2_9 | comp2332_c0_seq1 | 155 | Protein phosphatase 2C |  |
| 21_1 | comp8146_c0_seq1 | 60 | Calmodulin |  |
| 21_11 | comp19305_c0_seq1 | 189 | Serine/threonine protein kinase |  |
| 22_2 | comp1434_c0_seq3 | 190 | Calmodulin |  |
| 2_4 | comp203_c0_seq1 | 98 | 60S ribosomal protein L35 | Protein synthesis & metabolism |
| 21_7 | comp270_c0_seq1 | 109 | Ribosomal L9-like protein |  |
| 24_3 | comp2277_c0_seq1 | 233 | Ribosomal protein S6 kinase 2 alpha |  |
| 18_1 | comp130_c0_seq1 | 287 | Peroxidase 4 | Miscellaneous |
| 18_5 | comp160_c0_seq1 | 107 | Unknown Protein |  |
| 20_2 | comp2820_c0_seq1 | 155 | WD-40 repeat family protein |  |
| 22_4 | comp4682_c0_seq1 | 204 | Calcium-transporting ATPase 1 |  |
| 23_2 | comp3924_c0_seq1 | 104 | Unknown Protein |  |
| 3_2 | comp2249_c0_seq1 | 91 | Unknown Protein |  |
| 4_1 | comp4622_c0_seq1 | 116 | Nodal modulator 3 |  |
| 5_1 | comp1799_c0_seq1 | 131 | Cysteine proteinase inhibitor |  |
| 9_2 | comp4585_c0_seq1 | 95 | High affinity copper uptake protein |  |
| 9_6 | comp278_c0_seq1 | 219 | Farnesyl pyrophosphate synthase |  |
| 5_6 | n.a. | 112 | Wound induced protein |  |
| 19_1 | n.a. | 222 | Unknown Protein | Not available |
| 19_7 | n.a. | 197 | Unknown Protein |  |
| 22_4 | comp4682_c0_seq1 | 216 | n.a. |  |
| 14_4 | n.a. | 67 | n.a. |  |
| 15_1 | n.a. | 91 | n.a. |  |
| 18_7 | n.a. | 91 | n.a. |  |
| 22_3 | n.a. | 34 | n.a. |  |
| 22_5 | n.a. | 197 | n.a. |  |
|  |  |  |  |  |
| **Down regulated TDFs** | |  |  |  |
|  |  |  |  |  |
| 15_9 | comp7317_c0_seq1 | 167 | Laccase-13 | Cell wall |
| 11_10 | n.a. | 182 | Laccase |  |
| 11_6 | comp138_c0_seq1 | 187 | Pectinesterase |  |
| 12_4 | comp274_c0_seq1 | 164 | Expansin protein |  |
| 9_3 | comp312_c0_seq1 | 262 | Pectinesterase |  |
| 5_5 | comp3717_c0_seq1 | 81 | BHLH transcription factor | Transcription regulation |
| 9_5 | comp603_c0_seq1 | 199 | Histone H2A |  |
| 18_10 | comp15317_c0_seq1 | 141 | Histone-lysine N-methyltransferase |  |
| 18_14 | comp2153_c0_seq1 | 244 | Auxin response factor 4 |  |
| 21_3 | comp3196_c0_seq1 | 66 | Thioredoxin y | Signalling |
| 12_3 | comp6249_c0_seq1 | 156 | Calcium-dependent protein kinase 2 |  |
| 13_10 | comp7381_c0_seq2 | 79 | Protein kinase-like protein |  |
| 16_8 | comp26171_c0_seq1 | 72 | Protein serine/threonine kinase |  |
| 17_4 | comp5360_c0_seq5 | 158 | RLK, Receptor like protein |  |
| 17_6 | comp2671_c0_seq1 | 264 | Calcium-dependent protein kinase 2 |  |
| 11_7 | comp7186_c0_seq2 | 137 | Peptidyl-prolyl cis-trans isomerase | Protein synthesis & metabolism |
| 2_3 | comp63_c0_seq2 | 322 | DNAJ chaperone |  |
| 20_1 | comp3962_c0_seq1 | 407 | Ribosomal protein S6 kinase alpha-3 |  |
| 20_3 | comp343_c0_seq1 | 68 | 60S ribosomal protein L7 |  |
| 22_1 | comp953_c0_seq1 | 187 | 40S ribosomal protein S24 |  |
| 10_2 | comp47_c0_seq1 | 241 | Aminotransferase-like protein | Miscellaneous |
| 11_2 | comp709_c0_seq1 | 92 | Unknown Protein |  |
| 15_2 | comp8620_c0_seq1 | 127 | Unknown Protein |  |
| 17_1 | comp2181_c0_seq1 | 177 | Protein transport protein sec31 |  |
| 16_2 | comp262_c0_seq1 | 160 | Cortical cell-delineating protein |  |
| 18_3 | comp16247_c0_seq1 | 192 | Uncharacterized plant-specific domain 01589 |  |
| 18_8 | comp4199_c0_seq1 | 366 | Cysteine protease inhibitor 8 |  |
| 19_2 | comp125_c0_seq1 | 582 | 2-oxoglutarate-dependent dioxygenase |  |
| 19_4 | comp11040_c0_seq1 | 101 | Glucose transporter 8 |  |
| 19_6 | comp4249_c0_seq1 | 231 | Unknown Protein |  |
| 2_1 | comp489_c0_seq1 | 91 | F1F0-ATPase inhibitor protein |  |
| 2_5 | comp859_c0_seq1 | 250 | Unknown Protein |  |
| 2_7 | comp233_c0_seq1 | 185 | Photosystem I reaction center subunit VI, chloroplastic |  |
| 2_8 | comp1713_c0_seq1 | 179 | Phosphomethylpyrimidine synthase |  |
| 20_4 | comp12224_c0_seq1 | 118 | EXS (ERD1/XPR1/SYG1) family protein |  |
| 22_6 | comp5303_c0_seq1 | 89 | 1-aminocyclopropane-1-carboxylate oxidase-like protein |  |
| 23_1 | comp2630_c0_seq1 | 200 | Polyphenol oxidase |  |
| 24_1 | comp2476_c0_seq1 | 230 | Indole-3-glycerol phosphate synthase-like |  |
| 4_6 | comp34_c0_seq1 | 126 | cold, circadian rhythm, and rna binding 2 |  |
| 9_4 | comp33_c0_seq1 | 336 | Metallothionein-like protein |  |
| 22_7 | n.a. | 133 | n.a. | Not available |
| 3_4 | n.a. | 70 | n.a. |  |
| 4_3 | n.a. | 63 | n.a. |  |
| 13_3 | n.a. | 140 | n.a. |  |
